# On Bromide of Potassium

**Published:** 1864-08

**Authors:** 


### ON BROMIDE OF POTASSIUM.

By Dr. Garrod, F. R. S.

On the first introduction of Bromide of Potassium, it was thought to be very analogous in its action to the Iodide, although somewhat less powerful; but little, in fact, was known about its powers. About nine years since I made some extensive trials of this medicine, chiefly in hospital practice, and found that, in certain cases of eruptions of the skin, as in syphilitic psoriasis, it acted as a curative agent, or, at least, patients when under its influence lost the affections under which they had been suffering. I was induced to give the bromide in these cases as the patients were intolerant of the action of the iodide. I discovered, likewise, that Bromide of Potassium, when pure, did not give rise to any of the symptoms to which the name of Iodism has been applied. I did, indeed, occasionally notice these symptoms, but this led me more carefully to examine the salts which had been dispensed; and it was ascertained that, with one or two exceptions, the bromide, as sold in London, contained notable quantities of the Iodide of Potassium. After this, I took precautions to have the bromide pure in all my observations upon its action, and the results I arrived at may be thus summed up:—

1. It produces none of the irritation of the mucous membranes of the nose and fauces—no coryza.

2. Some patients experience a peculiar sensation of dryness of the throat and neighboring parts.

3. When given in large medicinal doses, sleepiness or drowsiness, and dull headache were occasionally noticed.

4. When administered in very large amounts, some loss of power was noticed in the lower extremities, which passed off when the medicine was discontinued.

5. The therapeutic action was decidedly what may be termed alterative —that is, it relieved certain forms of chronic disease, as syphilitic skin affections.

6. No marked action was observed upon the skin or kidneys.

Soon after these observations had been made, Sir Charles Locock stated that he had found Bromide of Potassium useful in hysterical epilepsy, and in other nervous affections connected with uterine disturbance, and I was from this led to make further trials of the remedy, and have found that—

7. Bromide of Potassium exerts a most powerful influence on the generative organs, lowering their functions in a remarkable degree.

8. It is a remedy possessing most valuable powers in diseases dependent on, and accompanied by, excitement or over action of the generative organs; and hence it may be given with advantage in nymphomania, priapism, certain forms of menorrhagia, especially that occurring at the climacteric period; as likewise in nervous convulsive diseases dependent on uterine irritation; and lastly, in some ovarian tumors.

9. It appears to produce an anæsthetic condition of the larynx and pharynx; and hence has been usefully employed in examinations and operations of these parts.

Bromide of Ammonium has been lately proposed more especially for the production of the last named effects, but I am not aware that it possesses any powers superior to those of the salt of potassium. The bromide of potassium may be given in doses of from five grains to ten or even fifteen grains to the adult.

It is curious to observe and compare the physiological and therapeutic powers of three salts so analogous to each other in a chemical point of view—namely, the Chloride, Bromide, and Iodide of Potassium, the first producing but little action unless given in large quantities, probably from its being a normal constituent of the body; the second, the bromide, abnormal to the economy, or existing only in infinitesimal amounts, acting especially on the nervous system; the third, the iodide, also abnormal to the body, having its influence more especially directed to the mucous membranes and secreting organs. The investigation of such actions in relation to the composition of the substances administered may probably one day afford some clue to the comprehension of the effects of remedies.—*Medical Times and Gazette, March* 12, 1864, *p.* 276—*Braithwaite's Retrospect.*

